# Development of SSR markers in *Paeonia* based on *De Novo* transcriptomic assemblies

**DOI:** 10.1371/journal.pone.0227794

**Published:** 2020-01-30

**Authors:** Dan He, Jiaorui Zhang, Xuefeng Zhang, Songlin He, Dongbo Xie, Yang Liu, Chaomei Li, Zheng Wang, Yiping Liu

**Affiliations:** 1 College of Forestry, Henan Agricultural University, Zhengzhou, Henan, China; 2 Henan Institute of Science and Technology, Postdoctor Research Base, Xinxiang, Henan, China; 3 Innovation Platform of Molecular Biology, College of Forestry, Henan Agricultural University, Zhengzhou, Henan, China; 4 College of Horticulture, Nanjing Agricultural University, Nanjing, Jiangsu, China; 5 Henan Institute of Science and Technology, Xinxiang, Henan, China; 6 Department of Genetics, Cell Biology, and Development, University of Minnesota, St Paul, Minnesota, United States of America; National Cheng Kung University, TAIWAN

## Abstract

Peony is a famous ornamental and medicinal plant in China, and peony hybrid breeding is an important means of germplasm innovation. However, research on the genome of this species is limited, thereby hindering the genetic and breeding research on peony. In the present study, simple sequence repeat (SSR) locus analysis was performed on expressed sequence tags obtained by the transcriptome sequencing of *Paeonia* using Microsatellite software. Primers with polymorphism were obtained via polymerase chain reaction amplification and electrophoresis. As a result, a total of 86,195 unigenes were obtained by assembling the transcriptome data of *Paeonia*. Functional annotations were obtained in seven functional databases including 49,172 (Non-Redundant Protein Sequence Database: 57.05%), 38,352 (Nucleotide Sequence Database: 44.49%), 36,477 (Swiss Prot: 42.32%), 38,905 (Clusters of Orthologous Groups for Eukaryotic Complete Genomes: 45.14%), 37,993 (Kyoto Encyclopedia of Genes and Genomes: 44.08%), 26,832 (Gene Ontology: 31.13%) and 37,758 (Pfam: 43.81%) unigenes. Meanwhile, 21,998 SSR loci were distributed in 17,567 unigenes containing SSR sequences, and the SSR distribution frequency was 25.52%, with an average of one SSR sequence per 4.66 kb. Mononucleotide, dinucleotide, and trinucleotide were the main repeat types, accounting for 55.74%, 25.58%, and 13.21% of the total repeat times, respectively. Forty-five pairs of the 100 pairs of primers selected randomly could amplify clear polymorphic bands. The polymorphic primers of these 45 pairs were used to cluster and analyze 16 species of peony. The new SSR molecular markers can be useful for the study of genetic diversity and marker-assisted breeding of peony.

## Introduction

Peony, which is a deciduous woody plant that belongs to genus *Paeonia*, is one of the traditional famous flowers in China. Peony had been cultivated for more than 1,600 years as an ornamental plant with high ornamental, edible and medicinal values. *Paeonia* is divided into three sections: *Paeonia*, *Moutan*, and *Onaepia*. Hybridization, as an important means of peony germplasm innovation, is the source of many excellent varieties of peony [1]. The offspring obtained from the distant hybridization between sect. *Paeonia* and sect. *Moutan* shows obvious heterosis. ‘Itoh’ known as the future of *Paeonia*, is the most famous hybrid with rich color, long flowering period, and vigorous growth [2]. However, in China, the distant hybridization of *Paeonia* is still in the primary stage. Although most of the interspecific distant hybridization of *Paeonia* can complete fertilization, embryos in the developmental process often shrink or die, and even individual full hybrids cannot develop into normal seedlings [3]. Embryo abortion can be caused by the abnormal development of embryo sac and endosperm, genetic incoordination between hybrid embryo, and endosperm, gene regulation and endogenous hormone regulation [4]. In recent years, many studies on embryo abortion after hybridization among peony have been reported. The Hybridization between *P*. ostii ‘Fengdanbai’ and *P*. *veitchiias* parents was conducted, and the result indicated that the free nuclear endosperm of aborted breeding embryo can not develop normally to provide nutrition for the original embryo [5]. However, only limited research has been done on the molecular mechanism of embryo abortion, and relatively complete genomic studies are lacking at present.

Transcriptome refers to the collection of all RNA, including mRNA and non-coding RNA, transcribed by cells or tissues of an organism in a specific state. Genes are annotated and labeled, through RNA-sequencing (RNA-seq) to analyze the expression and function of genes in different organisms [6]. The transcriptomic analysis of different tree peony organs, such as carpel, floral organ, and seed were reported. A comparative transcriptome was performed between two cultivars of *P*. *rockii* with different development patterns of carpel. From the transcriptome data, a total of 66,563 unigenes and 28,155 differentially expressed genes (DEGs) were identified. Among these DEGs, the genes *PsMYB114-like*, *PsMYB12*, and *PsMYB61-like* from the MYB gene family were probably the main characters that regulated the carpel quantitative variation [7]. A total of 29,275 unigenes were obtained from the bud transcriptome of tree peony. Among the DEGs, 64 flowering-related genes, and the genes of *PsAP1*, *PsCOL1*, *PsCRY1*, *PsCRY2*, *PsFT*, *PsLFY*, *PsLHY*, *PsGI*, *PsSOC1*, and *PsVIN3* probably regulated re-blooming [8]. A large number of DEGs on the transcriptome data of tree peony (*P*. *ostii*) seeds were related to oil biosynthesis and fatty acid metabolism [9]. In the present study, RNA-seq was used to discover the related pathways and important genes involved in the hybridization process of *Paeonia*.

Simple sequence repeat (SSR) is a widely distributed nucleotide sequence in the eukaryotic genome. SSR also has the characteristics of repeated occurrence and short sequence. In addition, SSR is polymorphic because of the different number of repeat sequences. Moreover, SSR is one of the most effective molecular markers among numerous molecular markers due to reproducibility, multi-allelic nature, high stability, relative abundance, co-dominant inheritance and good genome coverage [10, 11]. SSR loci are uniformly distributed in the entire genome of eukaryotes. The mutation frequency of SSRs is 30 times higher than the general random mutation, providing a good choice for the construction of plant gene map, genotype analysis and the study of genetic diversity. SSR markers are generally divided into genomic SSRs (G-SSRs) and expressed sequence tags (EST-SSRs) according to their position in the genome. EST-SSRs are more conservative than G-SSRs [12–14]. EST-SSR molecular markers have been widely used in the genetic diversity, genetic linkage map, and genome-wide association study analyses of many plants. The first high-density genetic linkage map of peony was constructed by using EST-SSRs [15]. A total of 2,253 potential SSRs were detected from 1,969 unigenes in the transcriptional sequencing analysis of tree peony flower buds, and 17,705 SSR motifs distributed in 13,797 sequences were obtained from the transcriptome of tree peony’s underground renewal buds [16, 17]. The detected EST-SSR markers were used in genetic diversity evaluation. The distribution frequency and regulation of SSR in the EST sequence of *Phalaenopsis aphrodite* were analyzed, and the polymorphism and universality of the SSR were evaluated [18].

Peony has a long history of cultivation and a complex genetic background. Reliable and effective molecular markers are needed for the analysis of the relationship among peony species, identification of hybrid seedlings, establishment of genetic map, and marker-assisted breeding [19]. SSR is one of the most useful molecular markers among many molecular markers, but SSR is rarely used in peony due to the insufficient number of SSR markers. Therefore, developing a large number of SSR molecular markers is urgently needed for peony research. In the present study, 21,998 SSR loci were identified, and 45 pairs of SSR primers were developed on the basis of the transcriptome sequence of the hybrid embryos of *Paeonia* to analyze the genetic diversity of peony germplasm, which provides a theoretical basis for the research on the abortion of hybrid embryos.

## Materials and methods

### Plant materials

Hybrid embryos were obtained from the hybridization of *P*. *ostii ‘*Fengdan*’* and *P*.*lactiflora* 'Fenyunu' from Henan Agricultural University with evident abortion differences in three different periods (pre-stage, mid-stage and late-stage). Three individual embryos from each stage were collected and pooled for each sample, and this collection was repeated three times to provide biological replicates. RNA extraction was conducted after mixed sampling as further research materials.

**Fig 1:**
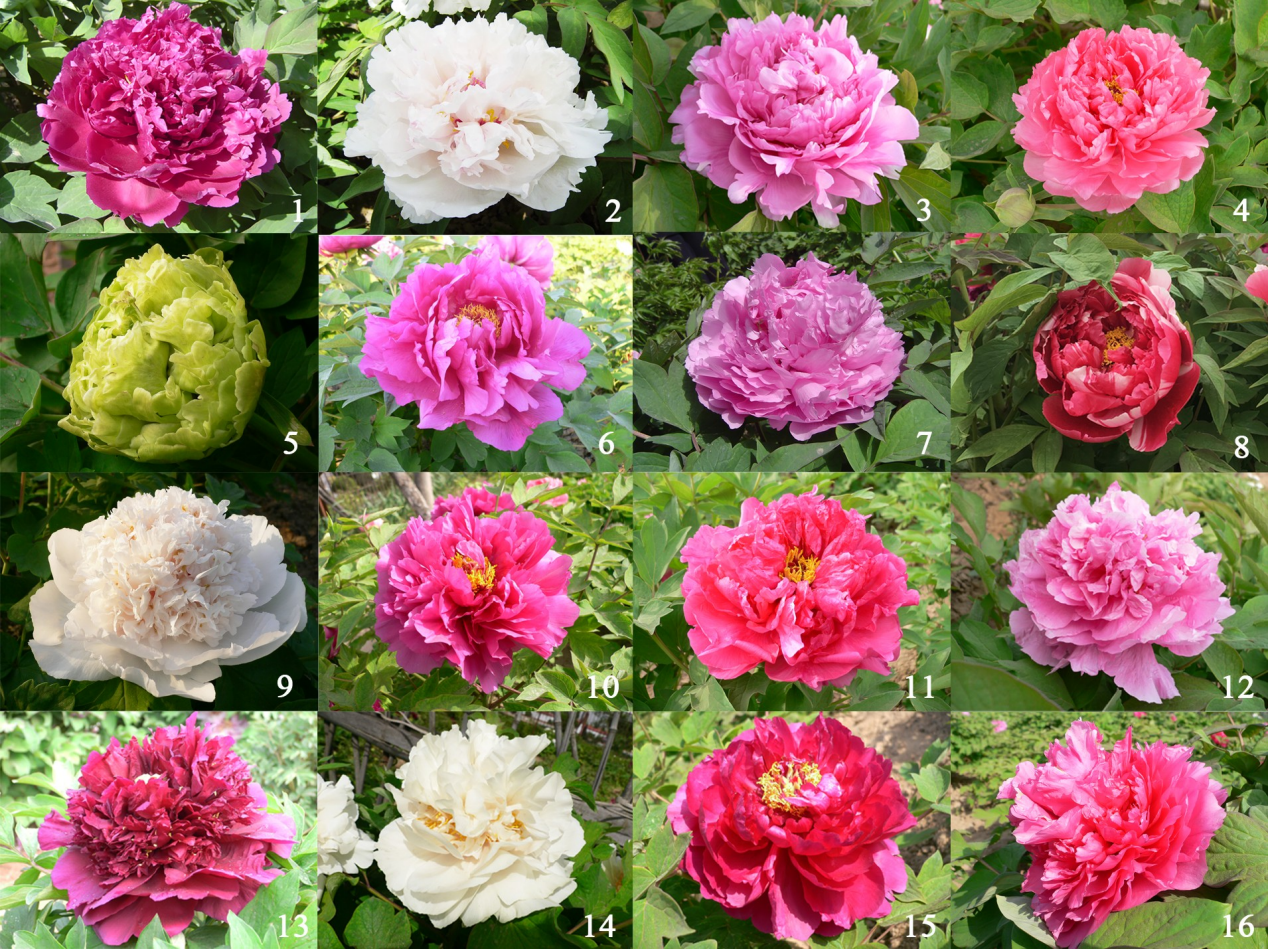
The flowers of 16 peony materials. 1-YLBZ, 2-JY, 3-SBH, 4-FGMT, 5-DL, 6-MJH, 7-SGJ, 8-EQ, 9-QXB, 10-LYH, 11-HBS, 12-CH, 13-YLZZP, 14-YH, 15-WLYH, 16-JYH.

The 16 peony materials for screening polymorphic markers were provided by Zhengzhou Botanical Garden (Table 1, Fig 1). Three or four leaves of each species were harvested, immediately frozen in liquid nitrogen and placed at −80°C for DNA extraction.

**Table 1:** Detailed information of *P*. *suffruticosa* in this study.

Number	Name of cultivar	Abbreviation	Flower pattern	Colours	Origin
1	Yinlin Bizhu	YLBZ	Crown form	Light purple	Heze of China
2	Jingyu	JY	Crown form	White	Heze of China
3	Shiba Hao	SBH	Prolification	Amaranth	Heze of China
4	Fugui Mantang	FGMT	Prolification	Pink	Heze of China
5	Doulv	DL	Globular form	Green	Luoyang of China
6	Manjiang Hong	MJH	Chrysanthemum form	Red	Heze of China
7	Shengge Jin	SGJ	Prolification	Amaranth	Heze of China
8	Erqiao	EQ	Rose form	Multicolor	Luoyang of China
9	Qingxiang Bai	QXB	Crown form	White	Heze of China
10	Luoyang Hong	LYH	Rose form	Amaranth	Luoyang of China
11	Hongbao Shi	HBS	Simple forme	Amaranth	Heze of China
12	Caihui	CH	Rose form	Pink	Heze of China
13	Yanlong Zizhu Pan	YLZZP	Crown form	Violet dark	Heze of China
14	Yaohuang	YH	Crown form	Light green	Luoyang of China
15	Wulong Yaohui	WLYH	Prolification	Amaranth	Heze of China
16	Juanye Hong	JYH	Prolification	Red	Heze of China

### Method

#### DNA and RNA extraction

Genomic DNA was extracted using a DNA extraction kit (DP301) of Tiangen Biotech (Co., Ltd., Beijing). DNA quality was detected in 1% agarose gel electrophoresis.

RNA was extracted using a total RNA extraction kit (DP441) of Tiangen Biotech (Co., Ltd., Beijing), and the concentration and quality of RNA were investigated by the NanoDrop ND-5000 Ultraviolet-visible spectrophotometer produced by Thermo Company. Qualified RNA was sent to BGI Genomics (Wuhan) for library construction, and three biological replicates and technique replicates were used in high-throughput the next-generation sequencing conducted on the BGISEQ -500 platform.

#### Transciptome analysis

Raw data were filtered using SOAPnuke software, and reads with joint contamination, low quality, and unknown bases were removed. De novo transcriptome was assembled using Trinity software. Assembled transcripts were clustered, and redundancy was removed to form non-redundant unigenes.

#### Functional annotation of unigenes

All the unigene sequences obtained from the three sequencing libraries were compared with the seven databases, including Non-Redundant Protein Sequence Database (NR), Nucleotide Sequence Database (NT), Gene Ontology (GO), Swiss-Prot, Kyoto Encyclopedia of Genes and Genomes (KEGG), Pfam, and Clusters of Orthologous Groups for Eukaryotic Complete Genomes (KOG) databases, by using Blastx software to obtain the functional annotation, biological process, and metabolic pathway information of relevant unigenes.

#### Identification of SSR loci and design of SSR primers

SSR loci from transcriptome sequence were identified using the Microsatellite (MISA) software (Microsatellite, http://pgrc.ipk-gatersleben.de/misa/). One to six nucleotides minimum repeats of 12, 6, 5, 5, 5 and 5 times were used as the identification criteria. The number and proportion of SSR types in *Paeonia* transcriptome and the frequency of different repeat times of each repeat type were counted.

The primers of each unigene sequence containing SSR loci were designed using Primer 3.0 software (http://bioinfo.ut.ee/primer3). Default values were selected as software parameters. One hundred pairs of primers were randomly selected and synthesized by Sangon Biotech (Co. Ltd., Shanghai) for the analysis of polymorphism and universality of primers.

#### Selection, amplification and validation of SSR markers

Polymerase chain reaction (PCR) was performed at a volume of 10 μl containing 5 μl of 2 × Taq PCR Master Mix (Biomiga), 1 μl of template DNA, 0.5 μl of forward primer (10 ng /μl) and 0.5 μl of reverse primer (10 ng /μl), 3 μl ddH2O. Amplification was performed according to the following procedure: pre-degeneration at 94°C for 5 min, degeneration at 94°C for 30 s, annealing temperatures for 30 s (annealing temperatures were set according to each primer in the range of 50°C—65°C), 40 cycles of 72°C extension for 40 s, and final extension 72°C for 10 min. The PCR products were processed in 1% agarose gel for 50 min at 90 v, and SSR primers with good stability were screened out.

Primers with good stability were used for PCR amplification in the SSR fluorescence primer reaction system. Fluorescent PCR was performed at a volume of 10 μl that contained of 5 μl of 2× Taq PCR Master Mix (Biomiga), 2 μl of template DNA (50 ng /μl), 0.15 μl forward primers (10 ng /μl) and 0.3 μl of reverse primer (10 ng /μl), 2.55 μl ddH2O. Forward primers were synthesized with an additional 18 nucleotides from the M13 universal primer appended to the 5′ end. The fluorescence amplification procedure was the same as the ordinary amplification procedure. PCR amplification products (0.5 μl) with fluorescence labeling were pooled and combined with Hi-Di formamide (9 μl) and LIZ-500 size standard (0.5 μl) (Applied Biosystems, USA) and analyzed using an ABI3730xl DNA Analyzer (Applied Biosystems, USA). Polymorphic locus information was identified through Genographer v2.1 software (http://sourceforge.net/projects/genographer).

#### Genetic diversity analysis

Sixteen peony cultivars were analyzed using SSR primers with polymorphism. Raw data, including polymorphic information content (PIC), observed number of alleles (Na), effective number of alleles (Ne), heterozygosities (Ho), expected heterozygosities (He), and Shannon’s diversity index (I) were analyzed using POPgene v1.32 software [20]. The genetic similarity of these materials was analyzed using NTSYS-PC software [21]. Based on these data, the cluster dendrogram of these varieties was drawn, and then the relationship among these varieties was analyzed.

## Results

### Sequencing and assembly

**Fig 2:**
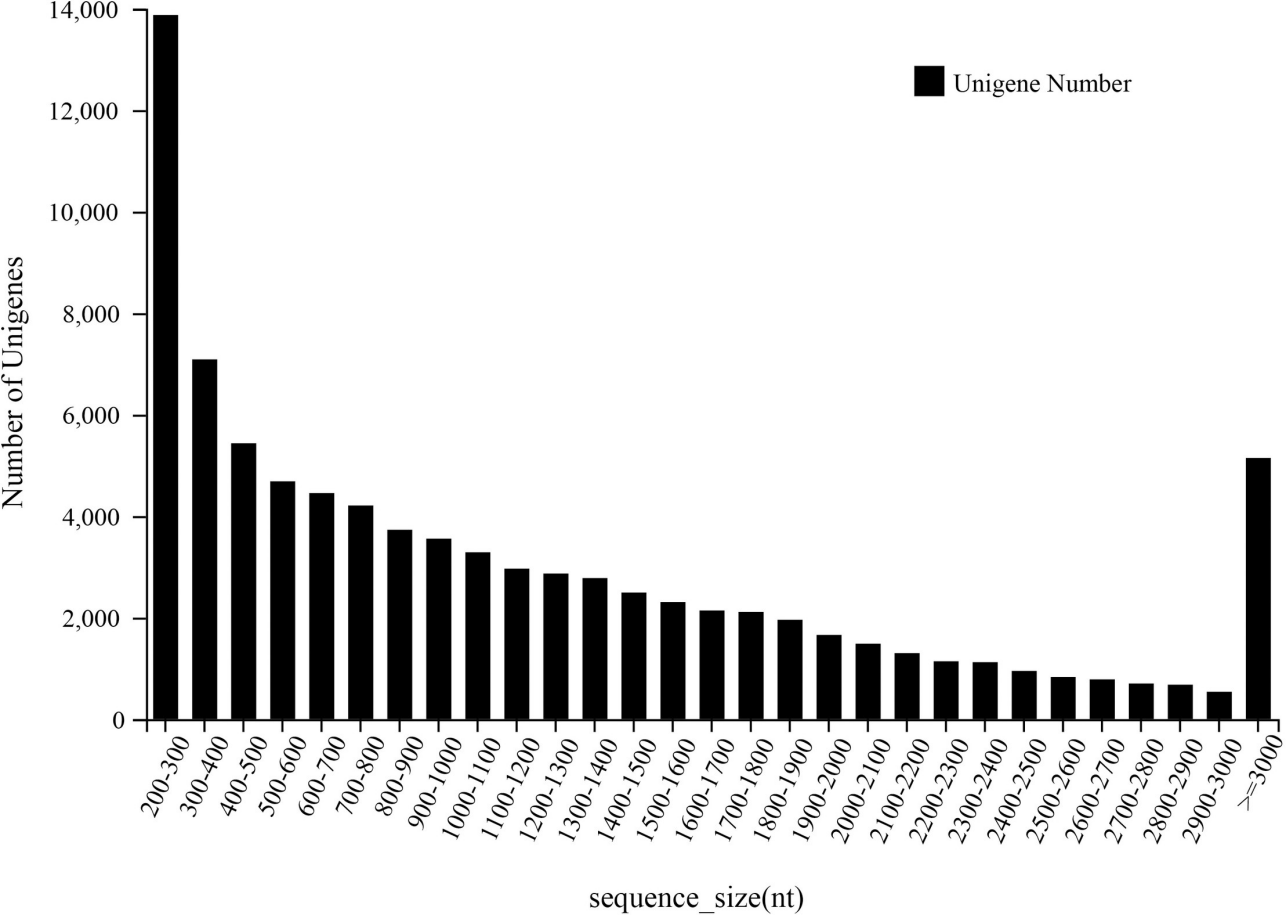
Length distribution of assembled unigenes from the transcriptome.

A total of 92.02 Gb of data were measured on the BGISEQ-500 platform, and 86,195 unigenes were obtained by assembling clean reads with Trinity. All data related to this study has been public available on NCBI with SRA accession PRJNA592882. The total transcriptome sequence length was 102,497,172 bp, average sequence length was 1,189 bp and N50 was 1,780 bp. A total of 20,957 unigenes (36.05%) had a sequence length of ≤ 600 bp. 15,939 unigenes (18.49%) had a length of 601–1000 bp. In addition, 24,539 unigenes (28.47%) had a length of 1001–2000 bp, and 14,642 unigenes (16.99%) had a sequence > 2000 bp. As a result, the number of unigenes assembled decreased as the sequence length of unigenes increased (Fig 2).

### Unigene annotation

All unigenes obtained by transcriptome sequencing were annotated into the KEGG, GO, NR, NT, Swiss-Prot, Pfam, and KOG databases. Finally, 49,172 (NR: 57.05%), 38,352 (NT: 44.49%), 36,477 (Swiss Prot: 42.32%), 38,905 (KOG: 45.14%), 37,993 (KEGG: 44.08%), 26,832 (GO: 31.13%), and 37,758 (Pfam: 43.81%) unigenes were functionally annotated, respectively.

**Fig 3:**
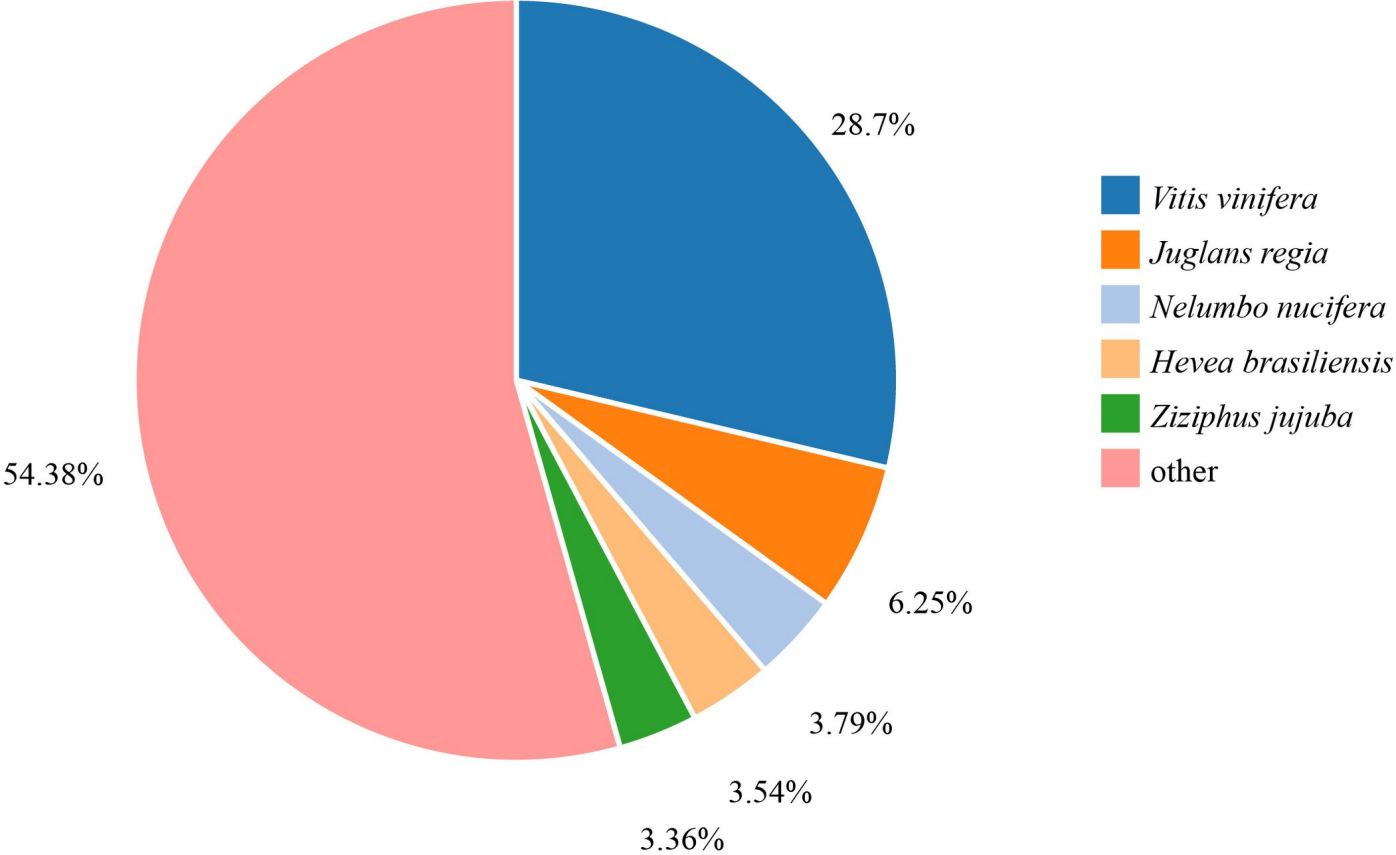
NR species distribution for the annotation with the NR database.

In the NR database, *Vitis vinifera* (28.7%) had the highest matching degree with the unigene sequence, followed by *Juglans regia* (6.25%), *Nelumbo nucifera* (3.79%), and *Hevea brasiliensis* (3.54%).*Ziziphus jujuba* had the lowest matching degree (3.36%), and 54.38% of the unigenes did not match the protein sequences of other species (Fig 3). Short unigene sequences are less likely annotated. In this study, the unigenes that did not match the protein sequences of other species were the unigenes (24.31%) with fragment length of 201–400 bp.

**Fig 4:**
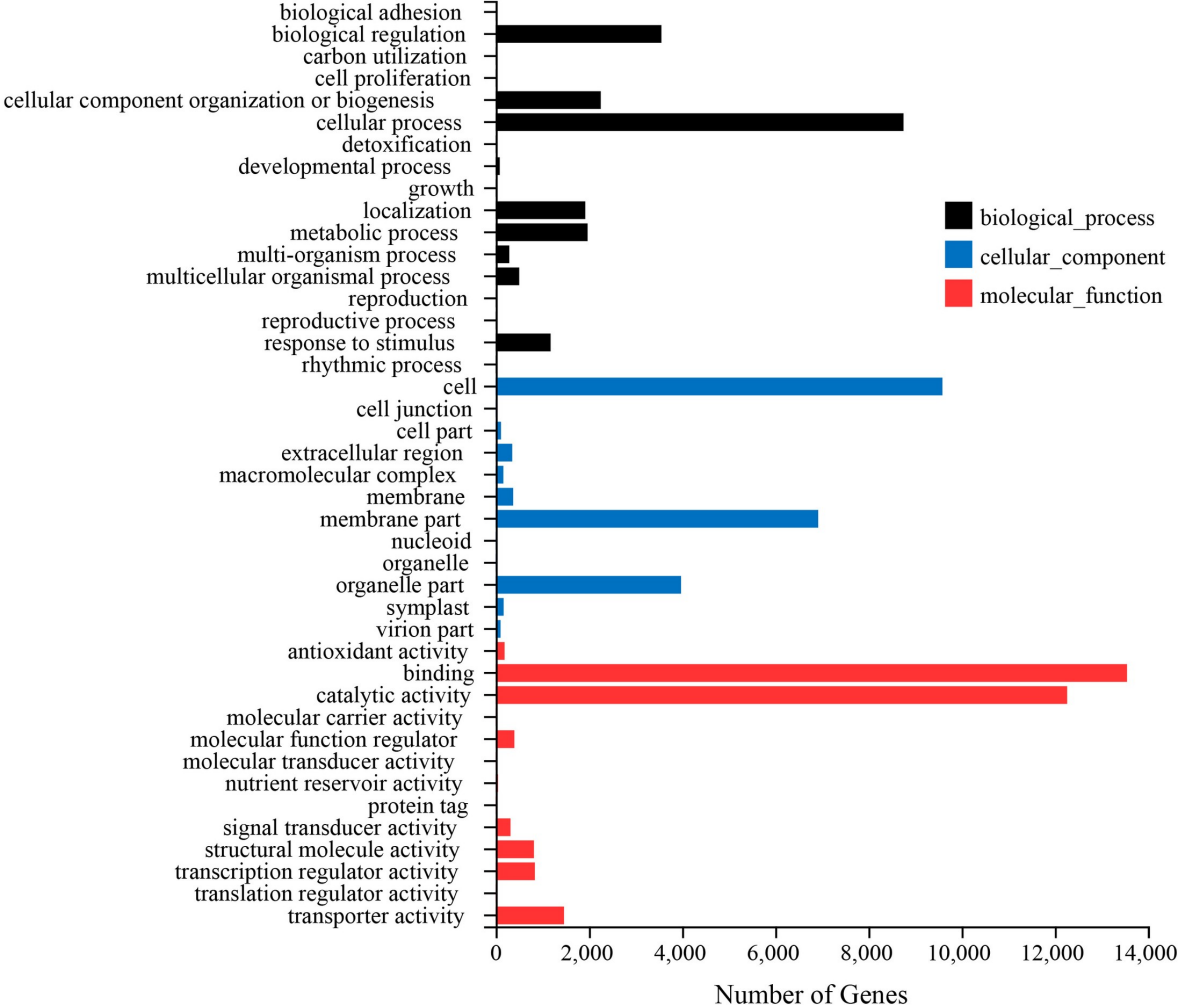
Functional annotations and classifications of unigenes in GO databases. GO analysis was performed at level 2 for the three main categories (biological process, cellular component and molecular function).

All unigenes compared with the NR database were further annotated into the GO database, and 26, 832 genes were annotated. GO database divided the unigenes into three functional categories including biological process, cell composition and molecular function. Seventeen types of genes including 15,509 unigenes were related to biological processes. Among them, 8,753 unigenes were involved in cellular processes, 3,557 unigenes were involved in biological regulation, 1,921 unigenes were involved in metabolic process, and 1,178 unigenes were involved in response to stimulus (Fig 4).

**Fig 5:**
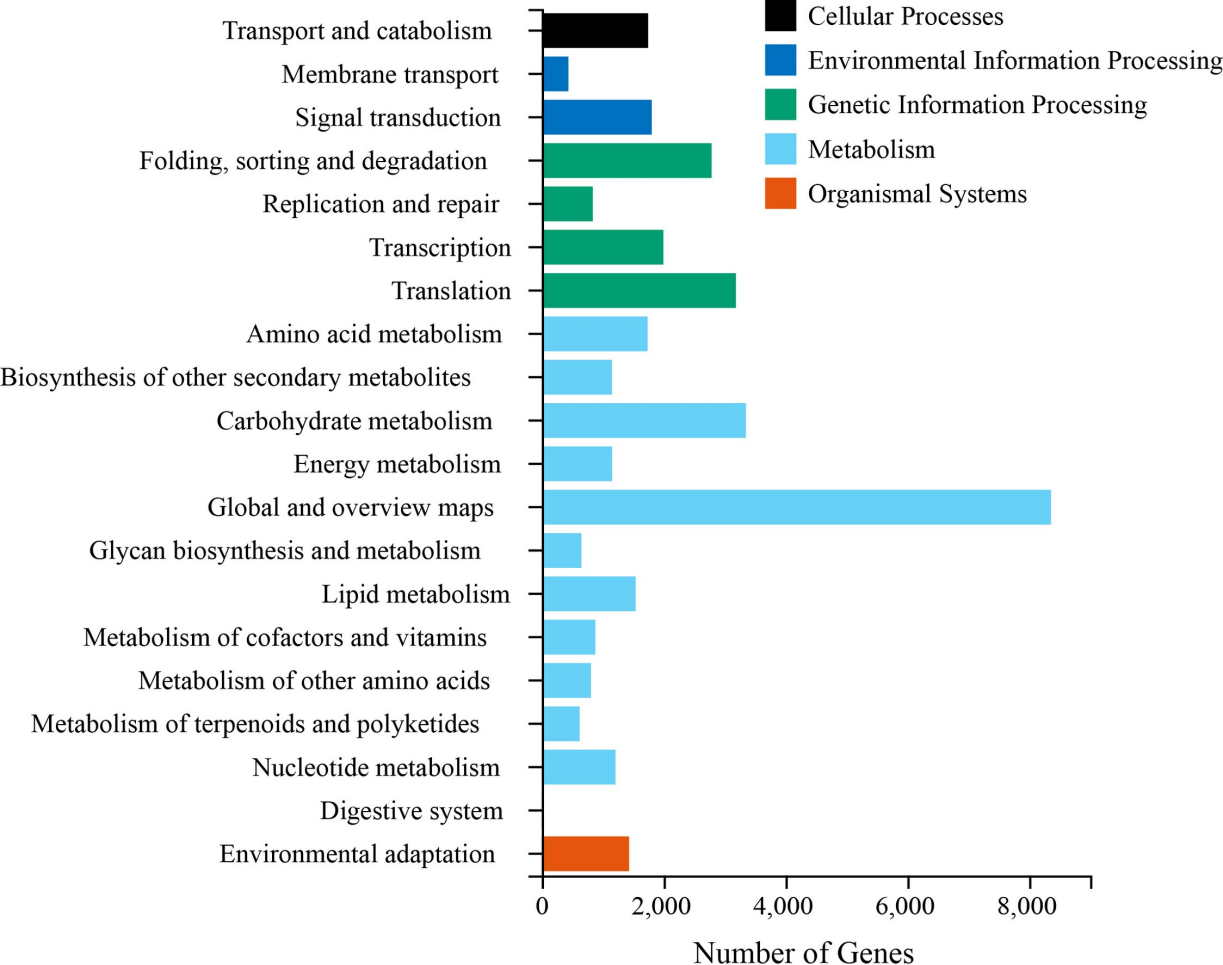
Functional annotations and classifications of unigenes in KEGG databases. KEGG analysis was performed at level 2 for five main categories (cellular processes, environmental information processing, genetic information processing, metabolism and organismal systems).

In the KEGG database, 37,993 unigenes were further annotated, analyzed, and classified into five major metabolic pathways: cell process, environmental information processing, genetic information processing, organismal systems and metabolism. Metabolism accounted for the largest proportion (56.46%) among the pathways. The five metabolic pathways were further divided into 20 subcategories. A maximum of 3,350 metabolites were related to carbohydrate metabolism; 3,186 metabolites were related to translation; 2,787 metabolites were related to folding, classification and degradation; 1,745 metabolites were related to transport and catabolism; and 1,737 metabolites were related to amino acid metabolism (Fig 5).

### Distribution and quantitative characteristics of SSR loci in peony transcriptome

SSR loci were detected in the assembled unigenes using MISA (http://pgrc.ipk-gaterslebende/misa/) software. The results showed that 21,998 SSRs were distributed in 17,567 unigenes, and the distribution frequency of SSR was 25.52%, with an average of one SSR per 4.66 kb. The main SSR motifs were mononucleotide, dinucleotide and trinucleotide, which account for 55.74%, 25.58%, and 13.21% of the total number of repeats, respectively (Table 2). In addition, tetranucleotide, pentnucleotide and hexonucleotide accounted for 0.65%, 1.32%, and 3.48% of the total number of repeats, respectively. The distribution frequency of SSR loci of different types of peony was consistent with their proportion. The repeat times of SSR motifs were distributed between 4 and 56 times, and the repeat times of mononucleotide were more than 12 times. The repeat times of dinucleotides were mainly 6, 7, and 8 times. The main repeat times in trinucleotides and tetranucleotides were 5 and 4 times respectively. Moreover, the main repeat times of pentnucleotides and hexonucleotides were 4 times.

**Table 2:** The number and distribution frequency of SSR types in *Paeonia*.

Type of repeat	Repeat times								Total	Ratio/%	Distribution Frequency/%
	4	5	6	7	8	9	10	>10			
Mononucleotide	\	\	\	\	\	\	\	12 262	12 262	55.74	14.22
Dinucleotide	\	\	1 820	1 122	830	529	354	973	5 628	25.59	6.53
Trinucleotide	\	1 806	573	299	140	25	20	43	2 906	13.21	3.37
Tetranucleotide	\	101	31	2	5	4	\	2	145	0.66	0.17
Pentanucleotide	239	43	5	3	\	1	\	\	291	1.32	0.34
Hexanucleotide	549	97	74	24	14	2	2	4	766	3.48	0.89
Total	788	2 047	2 503	1 450	989	561	376	13 284	21 998	100	25.52
Ratio/%	3.58	9.30	11.38	6.59	4.50	2.55	1.71	60.39			

### Screening of SSR primers and analysis of polymorphism

One hundred pairs of primers were randomly selected from the designed SSR primers for synthesis, and then 16 peony germplasm were amplified by RCR. Sixty pairs of primers capable of amplifying specific bands, among which 45 pairs showed good polymorphism, accounting for 45% of the total primers.

According to the analysis by POP gene software, 290 alleles were obtained by polymorphic primer amplification; with the distribution range of 2–11 alleles, and Na was 6.4444 (Table 3). SSR 43 was the primer that obtained the greatest number of alleles in the amplification. The mean value of the Ne of SSR locus was 3.9070, and the variation range was between 1.6187 and 7.0244. The mean value of Ho was 0.7785, and the variation range was 0.2667 to 1.0000. The mean value of He was 0.7302, and the variation range was 0.3954 to 0.9231. The average value of I was 1.4839, with a range of 0.6931 to 2.0786. The average value of PIC in this experiment was 0.6619, and the variation range was 0.3468 to 0.8418. The PIC values of SSR 23 and SSR 44 were the highest (0.8418), and the PIC values of SSR 33 were the lowest (0.3468).

**Table 3:** Statistical analyses of the genetic diversity of 45 SSR loci.

Locus	Na	Ne	Ho	He	I	PIC
SSR1	10	6.3380	0.5333	0.8713	2.0376	0.8159
SSR2	5	3.2267	0.6364	0.7229	1.3429	0.6427
SSR3	6	2.9412	0.8000	0.6828	1.3770	0.6265
SSR4	7	4.1667	1.0000	0.7862	1.6397	0.7299
SSR5	5	3.7190	0.9333	0.7563	1.4388	0.6891
SSS6	4	2.6806	0.9375	0.6472	1.1182	0.5559
SSR7	8	5.4217	0.8000	0.8437	1.8495	0.7922
SSR8	5	2.9138	1.0000	0.6831	1.2312	0.5932
SSR9	10	5.0562	0.9333	0.8299	1.9024	0.7788
SSR10	3	2.2727	1.0000	0.5793	0.8919	0.4613
SSR11	7	3.7692	0.8571	0.7619	1.5675	0.7007
SSR12	8	4.7368	1.0000	0.8161	1.7415	0.7584
SSR13	9	5.1136	0.9333	0.8322	1.8414	0.7779
SSR14	7	3.6835	1.0000	0.7520	1.5295	0.6886
SSR15	7	4.5000	0.8667	0.8046	1.6670	0.7452
SSR16	9	6.6977	0.9167	0.8877	2.0123	0.8329
SSR17	7	3.0625	0.7857	0.6984	1.4272	0.6333
SSR18	2	2.0000	1.0000	0.5172	0.6931	0.3750
SSR19	5	3.7815	0.9333	0.7609	1.4148	0.6881
SSR20	7	3.6056	0.6875	0.7460	1.5200	0.6795
SSR21	5	4.0179	0.9333	0.7770	1.4727	0.7085
SSR22	7	4.4554	1.0000	0.8023	1.6633	0.7432
SSR23	9	7.0244	0.9167	0.8949	2.0595	0.8418
SSR24	5	1.7193	0.3571	0.4339	0.8816	0.3966
SSR25	6	3.0866	0.3571	0.7011	1.3812	0.6361
SSR26	4	2.7778	0.6000	0.6621	1.1391	0.5789
SSR27	7	3.3835	0.8667	0.7287	1.4745	0.6606
SSR28	7	4.4118	0.7333	0.8000	1.6548	0.7402
SSR29	5	1.7928	0.2667	0.4575	0.9322	0.4205
SSR30	3	1.9310	0.4286	0.5000	0.7679	0.3954
SSR31	6	3.5714	0.8667	0.7448	1.4400	0.6728
SSR32	7	4.0000	0.6667	0.7826	1.6133	0.7147
SSR33	4	1.6187	0.3333	0.3954	0.7291	0.3468
SSR34	8	7.0000	0.5714	0.9231	2.0076	0.8406
SSR35	9	4.7368	0.8667	0.8161	1.8227	0.7644
SSR36	5	2.1127	0.6667	0.5448	1.0919	0.5005
SSR37	7	4.9231	0.7500	0.8226	1.7309	0.7687
SSR38	9	6.6275	0.5385	0.8831	2.0340	0.8328
SSR39	6	2.8800	0.7500	0.6812	1.3626	0.6193
SSR40	7	4.2453	0.9333	0.7908	1.6792	0.7366
SSR41	5	3.0118	0.8125	0.6895	1.2508	0.6121
SSR42	6	2.9091	0.5833	0.6848	1.3767	0.6250
SSR43	11	6.3210	0.7500	0.8690	2.0786	0.8247
SSR44	7	4.6667	0.9286	0.8148	1.7087	0.8418
SSR45	4	2.9032	1.0000	0.6782	1.1794	0.3966
Average	6.4444	3.9070	0.7785	0.7302	1.4839	0.6619

**Fig 6:**
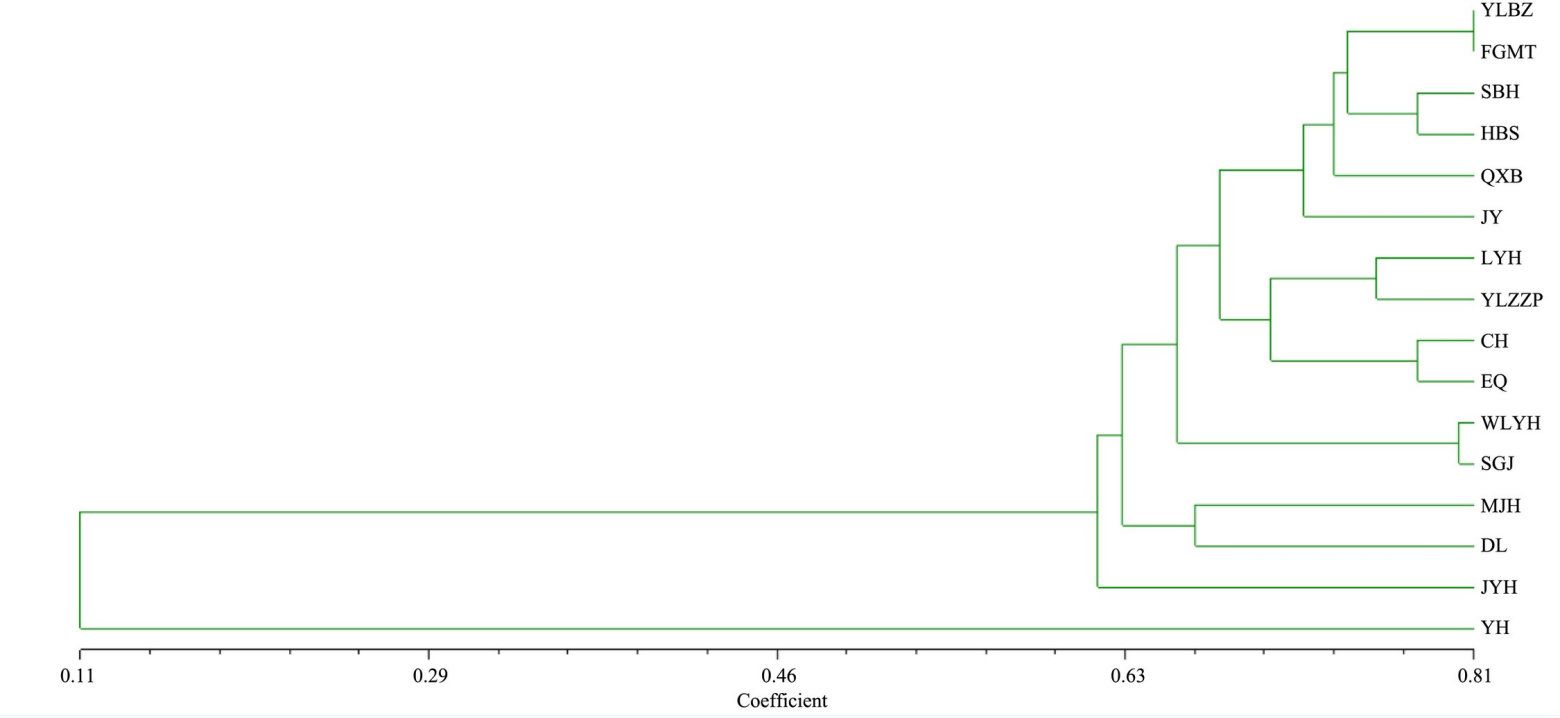
Dendrogram of 16 peony species revealed by the cluster analysis of the genetic similarity estimates generated by Nei coefficient based on 45 SSR markers.

NTSYS-PC software was used for the clustering analysis of 16 species of peony (Fig 6). The genetic similarity coefficient of the 16 peony species ranged from 0.11 to 0.81, and these germplasm were divided into two categories. ‘Yaohuang’ was a separate species, and the 15 other peony species were grouped. Such a group had a genetic similarity coefficient of 0.59, which indicated that ‘Yaohuang’ was far different from the other species. The genetic similarity coefficient of ‘Yinlin Bizhu’ and ‘Fugui Mantang’ was the highest at 0.81, indicating that the two were closely similar to each other. The next closest relatives were ‘Wulong Yaohui’ and ‘Shengge Jin’. ‘Jingyu’ and ‘Qingxiang Bai’ of the same color series and peony varieties of the same flower type were not grouped together.

## Discussion

### Transcriptome sequencing, assembly, and functional annotation

Peony is a non-model plant, and only little genetic information about peonies is available for research. The continuous development of new varieties makes the genetic diversity of its germplasm resources increasingly difficult to study. In this research, RNA-seq was used for investigating the peony transcriptome, and a total of 92.02 Gb of data were obtained. Taking N50 as an indicator to evaluate the assembly quality, the longer the length of N50 is within the range of 1000–2000 bp, the better the assembly quality can be [22]. In this study, N50 was 1,780 bp, indicating a good assembly. The average length of measured sequence obtained was 1,189 bp, which was higher than that of other tree peony researches [8, 17], *Syringa oblata* (853 bp) [23], flowering Chinese cabbage (779 bp) [24], and bamboo (736 bp) [25]. After assembly, a total of 86,195 unigenes were obtained, among which 49, 172 were annotated into the NR database. Among the homologous sequences of other species, the matching degree with *Vitis vinifera* was the highest. Transcriptome data annotated into the KEGG database showed that 37,993 unigenes were distributed in five categories of metabolic pathways. Among which, 3,350, 1737, 1,540, and 1,209 metabolites were related to carbohydrate metabolism, amino acid metabolism, lipid metabolism, and nucleotide metabolism. In this study, the abundant transcriptome information of peony was obtained through transcriptome sequencing, and the expressed genes were compared with various major databases for functional annotation, functional classification and metabolic pathway analysis. Hence, this study laid a theoretical foundation for the further screening of the key genes for embryo abortion in peony hybridization.

### Analysis of SSR markers in peony

In this study, 86,195 unigenes were searched for SSR loci based on the transcriptome sequence of *Paeonia*, and the results showed that 21,998 SSR loci were distributed in 17,567 unigenes. One SSR site was found per 4.66 kb on average, with a distribution frequency of 25.52%, which was higher than that of *Rhododendron fortune* (20.58%) [26] and *Sorbus pohuashanensis* (16.68%) [27], but lower than that of *Tagetes erecta* (28.29%) [28]. The results showed that the frequency and density of SSR site distribution in different types of plants were different, which might be caused by the differences in species, in the number and length of unigenes, in analysis tools and SSR site screening criteria.

In this study, the distribution of repeating motif types in the SSR loci of *Paeonia* was evidently unbalanced. Mononucleotide to trinucleotide were the main repetitive motifs, accounting for 94.53%, whereas tetranucleotide to hexanucleotide only accounted for 5.46% of the total. The partiality of these higher motifs was affected by the EST population size and was more likely limited by their own length. Except for mononucleotide, the frequency of dinucleotide was the highest, followed by that of trinucleotide. This result was similar to that of *Sorbus pohuashanensis* [27], and *Actinidia eriantha* [29], but different from that of *Hordeum vulgare* [30], flowering Chinese cabbage [24], and other plants, whose main type of SSR repeat motif was trinucleotide. In Gai’s research, dinucleotide motifs were found to be the most frequent motif type (62.78%) in *P*. *subaequalis*, followed by trinucleotide (35.61%), tetranucleotide (1.21%) and hexanucleotide (0.40%) [16]. In other peony studies, tri- and hexanucleotide repeats were found to be the most frequent motif types [12]. The proportion of SSR repeat motifs was different in various plant species probably due to the different evolutionary events experienced by different plant genomes [31].

In this study, 60 pairs of primers were used for the PCR amplification of 16 peony germplasm. Among these primers, 45 pairs showed polymorphism, accounting for 75% of the total primers, which was higher than other peony (47.3%) [12], *Sorbus pohuashanensis* (11.54%), [27] and *Rhododendron fortune* (71.11%) [26] but lower than *Tagetes erecta* (80.56%) [28]. The number of samples, source, genetic diversity, and other factors affected this ratio. The polymorphism ratio of peony was high, which provides an important basis for the subsequent use of these primers. Genetic diversity of peony germplasm was analyzed using 45 pairs of primers. PIC value is an index that indicates the information content of molecular markers, and is widely used in the evaluation of molecular marker polymorphism. PIC value > 0.5 indicates that the primer has high polymorphism, PIC value in the range of 0.25–0.5 indicates moderate polymorphism, and PIC value < 0.25 indicates low polymorphism. In this study, 38 of the 45 pairs (84%) of primers showed high polymorphism, and the rest showed moderate polymorphism. The highest PIC value was 0.8418, and the average value was 0.6619, indicating that the molecular markers of peony were rich in information and highly recognizable. Among the 45 pairs of primers, the number of alleles was 290, Na was 6.4444, Ho was 0.7785, and He was 0.7302, and the I was 1.4839. An et al. used 15 pairs of primers to amplify 65 polymorphic loci in 57 samples of *P*. *rockii*, which has an average number of alleles of 4.333 at each locus, an average PIC of 0.618, and an average He of 0.671 [32]. Yu et al. amplified 42 polymorphic loci from 48 peony samples with 12 pairs of primers, and found that the average PIC was 0.468, average Ho and He were 0.785 and 0.541, respectively, and the average I was 0.906 [33]. The experimental results showed that different cultivars of peony had differences in various genetic parameters. Nevertheless, the polymorphism information content, allele number, Shannon’s diversity index, and other indicators revealed that peony was rich in genetic diversity. Therefore, the 45 pairs of polymorphic primers in this study were feasible for the study on the genetic diversity of peony.

In this research, the classification of the genetic clustering of peony varieties had no significant relationship with the flower type and color of peony. The peony used here was different from the previous classification of peony varieties with flower type and color as the first and second level criteria. ‘Wulong Yaohui’ and ‘Shengge Jin’ were closely clustered probably because they have the same color and flower type. ‘Manjiang Hong’ and ‘Doulv’ were grouped together but have different color and flower type. The incongruence between the dendrogram and the division of cultivars might be due to the unclear genetic background of Chinese peony cultivars, the limited number of samples and markers, and the environment [12]. The genome of peony is complex, and the location of gene variation is uncertain. Hence, the genetic diversity of peony must be further studied.

## Conclusions

In conclusion, using transcriptome sequences is a convenient way to identify SSR markers in tree peony. In this study, RNA-seq was used to assemble the transcriptome data of peony, and a total of 86,195 unigenes were obtained. Finally, 49,172 (NR: 57.05%), 38,352 (NT: 44.49%), 36,477 (Swiss-Prot: 42.32%), 38, 905 (KOG: 45.14%), 37,993 (KEGG: 44.08%), 26,832 (GO: 31.13%), and 37,758 (Pfam: 43.81%) unigenes were annotated via functional annotation. The distribution frequency of SSR was 25.52%, with an average of one SSR sequence per 4.66 kb, and mononucleotide, dinucleotide, and trinucleotide were the main repeat types. Based on the above analysis results, the SSR primers of peony were developed, and 45 pairs of primers, which showed good polymorphism, were selected to amplify the polymorphic bands. These novel SSR markers have high polymorphism and transferability and thus represent a powerful tool for the genetic linkage map construction, germplasm identification, genetic diversity analysis, genetic improvement and molecular marker-assisted breeding of *Paeonia*.
